# Non-Dipping Pattern Is Associated with Periprocedural Myocardial Infarction in Hypertensive Patients Undergoing Elective Percutaneous Coronary Intervention

**DOI:** 10.3390/medicina61050794

**Published:** 2025-04-25

**Authors:** Ozkan Bekler, Alparslan Kurtul

**Affiliations:** 1 Department of Cardiology, Istanbul Medipol University, 34214 Istanbul, Turkey; 2 Department of Cardiology, Hatay Mustafa Kemal University, 31060 Hatay, Turkey; alparslan.kurtul@mku.edu.tr

**Keywords:** non-dipping, ambulatory blood pressure monitoring, percutaneous coronary intervention, periprocedural myocardial infarction

## Abstract

*Background and Objectives*: Non-dipping blood pressure (BP) patterns are associated with increased cardiovascular risk, but their role in periprocedural myocardial infarction (PMI) during elective percutaneous coronary intervention (PCI) remains insufficiently clarified. The objective was to investigate whether a non-dipping BP profile independently predicts PMI in hypertensive patients undergoing elective PCI. *Materials and Methods*: This prospective observational study enrolled 462 hypertensive patients undergoing elective PCI, categorized as dipping or non-dipping based on 24 h ambulatory BP monitoring (ABPM). Clinical, laboratory, and angiographic data were compared. PMI was defined according to the Fourth Universal Definition of Myocardial Infarction. Independent predictors of PMI were identified using multivariate logistic regression. *Results*: Of the 462 patients, 243 (52.6%) exhibited a non-dipping BP pattern. Non-dipping status was significantly associated with higher incidence of PMI (32.5% vs. 13.7%, *p* < 0.001) and a worse metabolic profile, including elevated blood glucose (*p* = 0.001), Hemoglobin A1c (*p* = 0.002), and white blood cell count (*p* = 0.001), and lower high-density lipoprotein cholesterol (*p* = 0.047). These patients more frequently underwent complex PCI (25.1% vs. 5.0%, *p* < 0.001). In multivariate analysis, the non-dipping BP pattern emerged as the strongest independent predictor of PMI (odds ratio 25.99, 95% confidence interval 3.16–213.92, *p* = 0.002), followed by complex PCI, number of stents, stent length, and diabetes mellitus. *Conclusions*: Non-dipping BP pattern is a powerful and independent predictor of PMI in hypertensive patients undergoing PCI. Incorporating ABPM into routine cardiovascular risk assessment may improve the identification of high-risk patients and allow for tailored preventive strategies.

## 1. Introduction

Arterial hypertension (HT) is one of the most prevalent and modifiable risk factors for cardiovascular diseases, particularly coronary artery disease (CAD) and myocardial infarction (MI) [1]. Among hypertensive individuals, the circadian variation in blood pressure (BP) plays a significant role in determining cardiovascular risk [2]. Based on ambulatory BP monitoring (ABPM), patients are classified as “dipping” if their nocturnal BP decreases by ≥10% and as “non-dipping” if this reduction is <10% [3]. A non-dipping BP pattern has been associated with increased cardiovascular morbidity, target organ damage, endothelial dysfunction, left ventricular hypertrophy (LVH), and higher overall mortality [4,5,6,7].

Percutaneous coronary intervention (PCI) is the most commonly employed revascularization strategy for patients with obstructive CAD [8]. However, it carries a non-negligible risk of periprocedural myocardial infarction (PMI), which is linked to adverse long-term outcomes. PMI can occur due to coronary microembolization, transient ischemia, microvascular dysfunction, or endothelial injury—all mechanisms that may be exacerbated in patients with poorly controlled or non-dipping HT [9].

Emerging evidence suggests that a non-dipping BP profile may contribute to increased sympathetic activity, impaired coronary vasodilation, increased arterial stiffness, and abnormal autonomic tone, thereby enhancing myocardial vulnerability during PCI. Previous studies have demonstrated that non-dipping BP patterns are associated with impaired coronary perfusion, increased silent myocardial ischemia, and adverse cardiovascular remodeling, particularly in hypertensive patients undergoing interventions [10,11,12]. However, the relationship between circadian BP variability and PMI incidence remains insufficiently investigated.

Therefore, this study aims to evaluate whether a non-dipping BP pattern is an independent predictor of PMI in hypertensive patients undergoing elective PCI. Understanding this association could enhance preprocedural risk stratification and inform individualized patient management strategies to reduce adverse outcomes.

## 2. Materials and Methods

### 2.1. Study Design and Population

**Figure 1:**
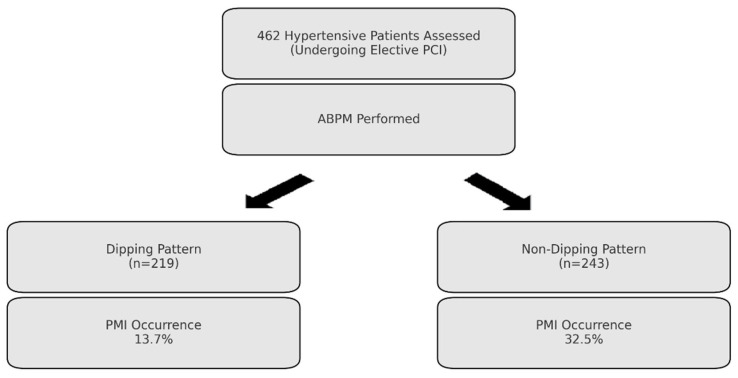
Flowchart of the study design and patient selection process.

This prospective single-center observational study included hypertensive patients who underwent elective diagnostic coronary angiography (CA) due to clinical indications, including symptoms of stable angina and abnormal stress test results, by either exercise treadmill tests or myocardial perfusion scintigraphy between January 2023 and December 2024. All procedures and assessments were performed under standardized institutional protocols by two experienced cardiologists, O.B. and A.K., in order to ensure assessment consistency throughout the study design. A flowchart outlining the study design and patient inclusion process is presented in Figure 1.

Inclusion criteria: Age ≥ 18 years, diagnosed hypertension, stable angina with confirmed CAD, and scheduled elective PCI. Exclusion criteria: Recent acute coronary syndrome (ACS) (<3 months), chronic kidney disease (estimated glomerular filtration rate [eGFR] < 30 mL/min), left ventricular (LV) systolic dysfunction (LV ejection fraction [LVEF] < 40%), significant valvular disease, arrhythmias affecting BP variability, or prior coronary artery bypass grafting (CABG) surgery.

### 2.2. Ambulatory Blood Pressure Monitoring (ABPM)

All patients underwent 24 h ABPM using an oscillometric device (e.g., Spacelabs 90207). Measurements were recorded every 15 min during the day (06:00–22:00) and every 15 min at night (22:00–06:00). Patients maintained normal daily activities while avoiding excessive exertion. ABPM assessments were performed within 1 to 3 days prior to the scheduled PCI procedure, in accordance with routine clinical practice. Antihypertensive medications were not stopped during the ABPM period. Classification was based on nocturnal BP reduction, with dipping showing a ≥10% decrease and non-dipping a <10% decrease. ABPM data were reviewed for accuracy [13].

### 2.3. Definition of Periprocedural Myocardial Infarction (PMI)

PMI was defined according to the Fourth Universal Definition of Myocardial Infarction (UDMI), requiring hs-cTnI elevation > 5× upper reference limit (URL) within 48 h post-PCI. For patients with elevated preprocedure high sensitivity-cardiac troponinI (hs-cTnI), PMI was confirmed if levels increased by >20% and exceeded 5× URL. Additional diagnostic criteria included new ischemic electrocardiography (ECG) changes, pathological Q waves, imaging-confirmed new regional wall motion abnormalities, or angiographic complications (dissection, occlusion, or embolization) [14].

To ensure accurate detection, hs-cTnI was measured pre-PCI and at 6, 12, 24, and 48 h post-PCI. Additional biomarker assessments were performed in symptomatic patients or those with significant ECG changes. These additional criteria were actively applied in clinical decision making, particularly in cases where biomarker values were borderline or when troponin elevation alone was insufficient to confirm PMI.

### 2.4. PCI Procedure

Indication for PCI was based on the presence of stable angina and angiographic evidence of significant CAD, defined as ≥50% luminal narrowing in the left main coronary artery (LMCA) and ≥70% stenosis in at least one major epicardial coronary artery with a reference vessel diameter > 2.5 mm [15]. The PCI procedures were performed either during the index procedure or on the following day/days after diagnostic CA. All PCI procedures were conducted by experienced interventional cardiologists following guideline-directed strategies. Vascular access was obtained via the radial or femoral artery, and anticoagulation was achieved with unfractionated heparin (70–100 IU/kg IV), with additional boluses given as needed. Lesion preparation and stent deployment were tailored to the lesion characteristics. Pre-dilation was performed in complex cases, and adjunctive techniques such as rotational atherectomy were used for calcified lesions. Post-dilation with non-compliant balloons was performed when necessary. All patients received dual antiplatelet therapy (DAPT) consisting of aspirin (75–100 mg daily) and clopidogrel (300–600 mg loading, 75 mg daily), initiated before PCI.

### 2.5. Definition of Complex PCI

Complex PCI was defined according to contemporary criteria as the presence of any of the following: implantation of ≥3 stents, treatment of ≥3 lesions, total stent length > 60 mm, bifurcation PCI requiring two stents, chronic total occlusion (CTO) intervention, or PCI involving unprotected LMCA. These criteria were in line with definitions used in prior studies assessing procedural risk and PMI occurrence [16].

### 2.6. Statistical Analysis

Statistical analyses were undertaken using SPSS V.21.0 software (SPSS Inc., Chicago, IL, USA). The normality of continuous variables was assessed using the Kolmogorov–Smirnov test. Continuous variables were compared using the independent samples *t*-test or Mann–Whitney U test (for not normally distributed variables), while categorical variables were analyzed using the chi-square test. Continuous data were presented as the mean ± standard deviation (SD) or median (interquartile range [IQR]), and categorical variables were presented as numbers and percentage of patients. Multivariate logistic regression analysis was conducted to identify independent predictors of PMI. All variables found to be statistically significant in univariate analysis, including non-dipping pattern, diabetes mellitus, number of stents, white blood cell (WBC) count, blood glucose, hemoglobin A1c (HbA1c), high-density lipoprotein (HDL) cholesterol, and SYNTAX score, were entered into the multivariate model. Relevant clinical comorbidities (e.g., diabetes mellitus, hyperlipidemia, and prior stroke) and cardiovascular medication use (e.g., beta-blockers, angiotensin converting enzyme inhibitors (ACE-I), angiotensin receptor bloclers (ARBs), statins, and antiplatelet agents) were included as covariates in both univariate and multivariate logistic regression models to control for potential confounding effects. The interaction terms, particularly between non-dipping pattern and diabetes mellitus and between non-dipping pattern and complex PCI, were explored in the statical analysis. These interaction terms were not statistically significant and therefore were not included in the final regression model. A two-sided *p*-value < 0.05 was considered statistically significant.

### 2.7. Ethical Considerations

The study was approved by the institutional ethics committee and conducted in accordance with the Declaration of Helsinki. Written informed consent was obtained from all participants.

## 3. Results

**Figure 2:**
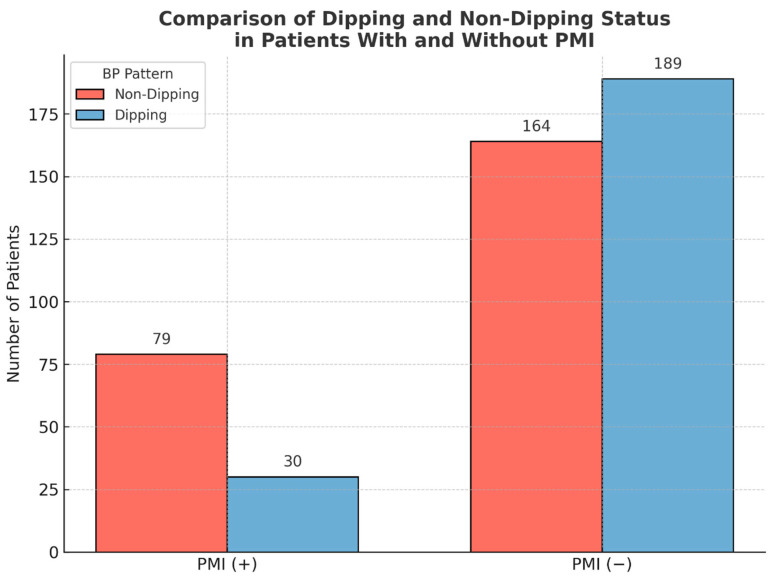
Comparison of PMI incidence between dipping and non-dipping hypertensive patients.

Among 462 hypertensive patients undergoing elective PCI, 243 (52.6%) demonstrated a non-dipping blood pressure pattern. Compared to the dipping group, non-dipping patients had significantly higher prevalence of diabetes mellitus (49.4% vs. 36.5%, *p* = 0.005), more frequent use of beta-blockers (63% vs. 51.1%, *p* = 0.01), and significantly more three-vessel disease (20.2% vs. 4.6%, *p* < 0.001). Non-dipping patients also exhibited increased WBC counts (9.53 ± 3.22 vs. 8.61 ± 2.28 × 10^9^/L, *p* = 0.001) and higher blood glucose (163.47 ± 82.79 vs. 136.15 ± 63.39 mg/dL, *p* = 0.001) and HbA1c levels (8.09 ± 2.45% vs. 6.21 ± 1.59%, *p* = 0.002), as well as higher creatinine levels (0.90 [0.77–1.00] vs. 0.85 [0.72–1.00] mg/dL, *p* = 0.045). Although non-dipping patients had lower HDL cholesterol (35.15 [32–44] vs. 39 [33–45] mg/dL) and LDL cholesterol (105.00 [81–137] vs. 105.80 [82–133.5] mg/dL) and higher triglyceride levels (168.25 [123.5–286.5] vs. 151 [109–239] mg/dL), these differences did not reach statistical significance. C-reactive protein (CRP) (5.12 [2.91–14.60] vs. 3.94 [2–10.85] mg/L) and platelet count (245 [198–291] vs. 238 [190–287] × 10^9^/L) were also similar between groups (*p* > 0.1). SYNTAX scores were significantly higher in the non-dipping group (11.25 [8–20.5] vs. 9 [6–13], *p* < 0.001). Complex PCI procedures were notably more frequent among non-dippers (25.1% vs. 5.0%, *p* < 0.001), while the prevalence of proximal lesions was comparable between groups (59.0% vs. 59.4%, *p* = 0.984). The incidence of PMI was significantly higher in non-dipping patients (32.5%) compared to dipping patients (13.7%, *p* < 0.001) (Table 1, Figure 2).

PMI occurred in 109 patients (23.6%) across the entire cohort. Compared with patients without PMI, those with PMI had a significantly higher prevalence of diabetes mellitus (52.3% vs. 40.5%, *p* = 0.030), non-dipping status (72.5% vs. 46.5%, *p* < 0.001), and three-vessel disease (25.7% vs. 8.8%, *p* < 0.001). The PMI group also exhibited significantly elevated WBC counts (9.63 ± 3.09 vs. 8.93 ± 2.76 × 10^9^/L, *p* = 0.037), total cholesterol (202.6 ± 72.44 vs. 174.85 ± 45.97 mg/dL, *p* = 0.008), blood glucose (166.71 ± 80.79 vs. 146.16 ± 73.39 mg/dL, *p* = 0.044), and HbA1c (8.93 ± 2.46% vs. 6.93 ± 2.12%, *p* = 0.003). Baseline renal functions were significantly lower in PMI patients, as reflected by the lower eGFR (82.36 ± 20.13 vs. 91.98 ± 21.94 mL/min/1.73 m^2^, *p* = 0.001) and higher creatinine levels (0.91 [0.79–1.01] vs. 0.88 [0.73–1.00] mg/dL, *p* = 0.017). HDL (34.5 [31–46] vs. 38 [33–44] mg/dL), LDL (99.75 [81–130] vs. 107 [82–134] mg/dL), and triglyceride levels (167 [135–287] vs. 155.5 [112–242] mg/dL) showed no statistically significant differences. Similarly, CRP (4.95 [3.02–10.85] vs. 4.89 [2.12–11.92] mg/L, *p* = 0.694) and platelet count (238.5 [195–273.5] vs. 244.5 [194.5–292] × 10^9^/L, *p* = 0.612) did not differ between groups. The procedural characteristics of PMI patients included more frequent complex PCI (40.4% vs. 7.8%, *p* < 0.001), increased number of stents (1.72 ± 0.74 vs. 1.27 ± 0.5, *p* < 0.001), greater total stent length (29.88 ± 10.2 vs. 24.93 ± 9.22 mm, *p* < 0.001), and more frequent proximal lesion involvement (73.4% vs. 54.5%, *p* = 0.001). Their SYNTAX score was also significantly higher (22.25 [14.5–28] vs. 9 [6–12], *p* < 0.001) (Table 2).

**Figure 3:**
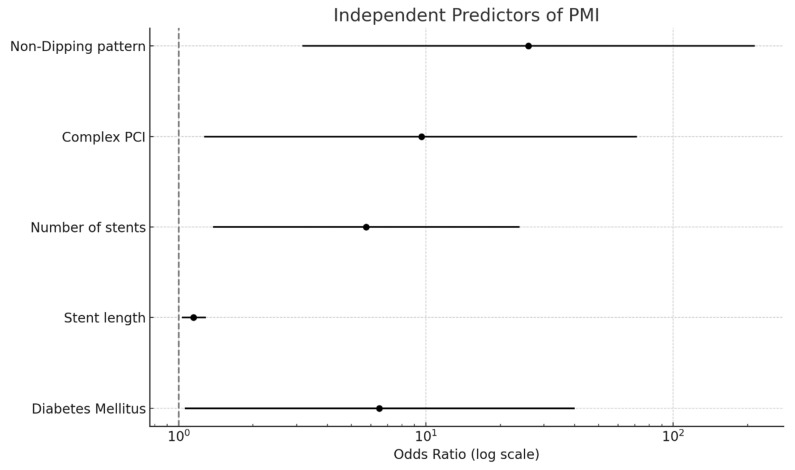
Independent predictors of PMI in hypertensive patients undergoing elective PCI.

In the multivariate logistic regression analysis, the non-dipping BP pattern emerged as the strongest independent predictor of PMI (odds ratio [OR]: 25.99; 95% confidence interval [CI]: 3.16–213.92; *p* = 0.002). Other independent predictors included complex PCI (OR: 9.62; 95% CI: 1.27–71.43; *p* = 0.029), number of stents (OR: 5.74; 95% CI: 1.38–23.96; *p* = 0.016), stent length (OR: 1.15 per mm increase; 95% CI: 1.03–1.29; *p* = 0.016), and diabetes mellitus (OR: 6.49; 95% CI: 1.06–40.00; *p* = 0.044) (Figure 3). Variables such as WBC count, creatinine, total cholesterol, and proximal lesion location did not remain statistically significant in the final model (Table 3).

## 4. Discussion

Our findings indicate that a non-dipping BP pattern is a strong and independent predictor of PMI in hypertensive patients undergoing elective PCI. The incidence of PMI was notably higher among non-dipping (32.5%) compared to dipping (13.7%) patterns, a difference that was both statistically and clinically significant (*p* < 0.001). Even after adjusting for established risk factors such as diabetes mellitus, stent burden, and procedural complexity, multivariate logistic regression identified non-dipping status as the most robust predictor. This emphasizes the growing recognition of circadian BP variability—measured through 24 h ABPM—as a powerful tool for preprocedural cardiovascular risk stratification, complementing anatomical and procedural risk indicators.

Numerous studies have shown that a non-dipping blood pressure profile is associated with adverse cardiovascular remodeling, such as LV hypertrophy, endothelial dysfunction, and increased arterial stiffness, all of which are known to impair myocardial perfusion and resilience during stress [17,18,19,20]. In our cohort, non-dipping patients also had a significantly higher prevalence of comorbidities, particularly diabetes mellitus and more frequent performance of complex PCI procedures involving longer stent lengths and multiple stent deployments. While these procedural and clinical factors are known contributors to PMI, the fact that a non-dipping status remained an independent predictor in multivariate analysis highlights its unique role in cardiovascular vulnerability. This supports the hypothesis that a non-dipping pattern reflects a distinct neurohumoral and vascular phenotype that is beyond the burden of conventional risk factors.

The elevated risk of PMI in non-dipping patients may be attributed to a constellation of hemodynamic, autonomic, and microvascular abnormalities. Unlike the physiological nocturnal decline observed in dipping, a non-dipping HT pattern is marked by persistently elevated nighttime BP, indicating a blunted autonomic modulation, particularly impaired vagal activity and exaggerated sympathetic tone during sleep [21,22,23]. This altered neuroautonomic state fosters increased myocardial oxygen consumption, reduced coronary vasodilatory capacity, and a proinflammatory vascular environment—all of which converge to exacerbate ischemic susceptibility during mechanical interventions such as PCI [24]. The resulting mismatch between oxygen supply and demand, especially under procedural stress, may render the myocardium more vulnerable to injury, even in the absence of overt epicardial obstruction.

In our study, WBC count was significantly higher in non-dipping hypertensive patients compared to dipping patients, consistent with the notion that non-dipping status is associated with a higher systemic inflammatory burden. However, WBC did not independently predict PMI in multivariate analysis. This suggests that while inflammation may be a feature of the non-dipping phenotype, it may not directly mediate acute ischemic injury during PCI. Similar findings have been reported by Tatsukawa et al., who showed that elevated WBC counts were associated with hypertension incidence but lost predictive strength after multivariate adjustment, particularly in time-fixed models [25]. In addition to inflammatory parameters, non-dipping status was also associated with metabolic dysregulation, including higher blood glucose and HbA1c levels. Although these variables were significantly elevated in non-dipping patients in univariate analysis, they did not remain independent predictors of PMI in the multivariate model. This suggests that adverse metabolic profiles may coexist with the non-dipping pattern without directly mediating periprocedural myocardial injury. These findings are supported by Manea et al., who demonstrated a high prevalence of non-dipping patterns in patients with type 2 diabetes and HT, yet they reported that glycemic markers, such as HbA1c, were not significant independent predictors after multivariate adjustment [26].

Beyond autonomic dysregulation, endothelial dysfunction emerges as a pivotal contributor to cardiovascular risk in non-dipping patients [27,28,29]. The lack of nocturnal BP dipping has been associated with impaired nitric oxide synthesis, enhanced oxidative stress, and elevated circulating inflammatory cytokines, all of which hinder endothelial-dependent vasodilation [30,31,32]. This dysfunction not only attenuates coronary flow reserve but also impairs arteriolar autoregulation, reducing the heart’s ability to adapt to increased oxygen demand. Notably, several studies have linked elevated TIMI frame counts and slow coronary flow in non-dipping pattern patients to microvascular dysfunction—even in patients with angiographically normal epicardial vessels [33,34]. Recent studies have also demonstrated that non-dipping hypertension is independently associated with a higher coronary artery calcium score, indicating a greater subclinical atherosclerotic burden in these patients [35,36]. During PCI, where coronary flow dynamics are transiently disrupted, such baseline microvascular impairment may predispose patients to distal embolization, ischemia–reperfusion injury, and consequently, PMI.

In addition, the higher prevalence of arterial stiffness and LV hypertrophy in non-dipping individuals may further compromise coronary perfusion, particularly during diastole, when most myocardial blood flow occurs [37,38,39]. Increased afterload and impaired ventricular relaxation can reduce subendocardial perfusion, promoting silent ischemia, which may remain clinically unrecognized until provoked by procedural stress. Several hemodynamic studies have revealed elevated TIMI frame counts and reduced coronary flow velocities in non-dipping hypertensive patients, consistent with microvascular rarefaction and dysfunction, even in the absence of significant epicardial stenosis [40,41,42,43]. These alterations may render myocardial tissue particularly vulnerable to minor procedural insults, leading to a higher incidence of biomarker-defined PMI.

Notably, the nocturnal hypertensive load may contribute to silent myocardial ischemia, a frequently overlooked but clinically significant phenomenon indicating subclinical myocardial stress [44,45]. In non-dipping patients, the persistent elevation in nighttime BP impairs coronary autoregulation and heightens susceptibility to even transient ischemic episodes. During PCI, events such as distal embolization, balloon inflation, or temporary flow limitation can reveal these latent vulnerabilities, triggering measurable cardiac troponin release consistent with PMI. Therefore, the non-dipping profile may not only represent a chronic maladaptive cardiovascular state but also serve as a dynamic marker of impaired myocardial resilience—particularly under procedural or ischemic challenge.

Although procedural factors such as the SYNTAX score, stent length, and stent count were also independently associated with PMI in our analysis, the non-dipping BP pattern demonstrated the highest predictive strength. This finding underscores a crucial shift in procedural risk assessment as follows: beyond anatomical and procedural complexity, dynamic hemodynamic parameters—specifically circadian blood pressure rhythms—must be considered in comprehensive preprocedural evaluation. The integration of 24 h ABPM into PCI planning could refine existing risk models by identifying high-risk individuals who may benefit from enhanced myocardial protection strategies, including tighter BP control, anti-ischemic therapy optimization, or even procedural modifications [46,47,48].

This study contributes novel insights to the existing literature by demonstrating that circadian BP patterns—specifically the non-dipping profile—have prognostic value not only for long-term cardiovascular outcomes but also for acute procedural events such as PMI. While 24 h ABPM is well-recognized for its utility in stratifying long-term risk in hypertensive patients, its application in periprocedural cardiovascular risk modeling remains underexplored. By highlighting the association between non-dipping status and increased PMI risk, our findings suggest that ABPM-derived metrics can serve as practical and accessible tools to guide individualized risk mitigation strategies before PCI. This bridges a critical gap between chronic hypertension management and acute procedural planning, potentially redefining how we assess vulnerability in cardiovascular interventions.

### 4.1. Clinical Implications and Future Directions

The clinical implications of these findings extend beyond procedural planning. The integration of ABPM into routine preprocedural evaluation may help identify hypertensive patients at elevated risk for PMI, enabling more personalized management strategies. To promote broader implementation, efforts should focus on increasing physician awareness, incorporating ABPM recommendations into clinical guidelines, and addressing systemic barriers such as device access and reimbursement. Although this study focused on hypertensive individuals, the pathophysiologic mechanisms underlying the non-dipping profile—autonomic dysregulation, inflammation, and microvascular dysfunction—may also be relevant to other high-risk populations, including those with diabetes or chronic kidney disease. Future research should also evaluate implementation strategies for ABPM in real-world cardiology settings.

### 4.2. Study Limitations

Despite its strengths, this study has certain limitations that warrant consideration. First, its single-center design and observational methodology may limit the generalizability of findings across broader, more diverse populations. Second, PMI diagnosis was primarily based on biochemical markers (hs-cTnI) in accordance with the Fourth UDMI; however, this may not comprehensively capture functional myocardial impairment or clinical outcomes. Third, although the non-dipping pattern emerged as a robust predictor of PMI, the causal mechanisms underlying this association remain speculative. Future studies incorporating advanced imaging modalities—such as cardiac magnetic resonance or myocardial perfusion SPECT/PET—could provide mechanistic insights into microvascular dysfunction and myocardial susceptibility. Finally, large-scale multicenter trials using advanced diagnostic tools are required to validate the prognostic value of non-dipping patterns and clarify underlying mechanisms.

## 5. Conclusions

In conclusion, a non-dipping BP pattern is a strong, independent predictor of PMI in hypertensive patients undergoing PCI. Incorporating circadian BP variability into routine preprocedural evaluation may enhance risk stratification and guide targeted preventive strategies.

## Figures and Tables

**Table 1:** Comparison of baseline clinical, demographic, and laboratory characteristics between dipping and non-dipping patients undergoing PCI.

	Non-Dipping Patients (n = 243)	Dipping Patients (n = 219)	*p*
Age (years)	61.35 ± 9.81	61.72 ± 9.35	0.680
Gender (male, %)	191 (78.6)	167 (76.3)	0.547
Diabetes mellitus (n, %)	120 (49.4)	80 (36.5)	0.005
Hyperlipidemia (n, %)	138 (56.8)	122 (55.7)	0.815
Active smoking (n, %)	103 (42.4)	92 (42)	0.935
Family history of CAD (n, %)	93 (38.3)	80 (36.5)	0.699
Stroke (n, %)	9 (3.7)	4 (1.8)	0.223
ACEi or ARB use (n, %)	131 (53.9)	113 (51.6)	0.619
Beta-blockers use (n, %)	153 (63)	112 (51.1)	0.01
Aspirin use (n, %)	144 (59.3)	114 (52.1)	0.119
Statin use (n, %)	77 (31.7)	67 (30.6)	0.8
CCB use (n, %)	86 (35.4)	74 (33.8)	0.718
Left ventricular ejection fraction (%)	55.86 ± 4.58	56.62 ± 4.49	0.086
Hemoglobin (g/dL)	13.21 ± 1.77	13.11 ± 2.12	0.605
White blood cell count (10^9^/L)	9.53 ± 3.22	8.61 ± 2.28	0.001
Platelet count (10^9^/L)	238 (190–287)	245 (198–291)	0.295
Baseline GFR (mL/min/1.73 m^2^)	88.30 ± 23.63	90.60 ± 19.14	0.392
Baseline creatinine (mg/dL)	0.85 (0.72–1.00)	0.90 (0.77–1.00)	0.045
Total cholesterol (mg/dL)	181.30 ± 51.16	175.09 ± 45.43	0.398
LDL cholesterol (mg/dL)	105.80 (82–133.5)	105.00 (81–137)	0.901
HDL cholesterol (mg/dL)	39 (33–45)	35.15 (32–44)	0.111
Triglyceride (mg/dL)	151 (109–239)	168.25 (123.5–286.5)	0.141
Glucose (mg/dL)	163.47 ± 82.79	136.15 ± 63.39	0.001
Hba1c %	8.09 ± 2.45	6.21 ± 1.59	0.002
C-reactive protein (mg/L)	3.94 (2–10.85)	5.12 (2.91–14.60)	0.109
SYNTAX Score	9 (6–13)	11.25 (8–20.5)	<0.001
Three-vessel disease (n, %)	49(20.2)	10(4.6)	<0.001
Complex PCI (n, %)	54(25.1)	7(5.04)	<0.001
Proximal lesion (n, %)	125(59)	101(59.4)	0.984
PMI (n, %)	79(32.5)	30(13.7)	<0.001

PCI: percutaneous coronary intervention; CAD: coronary artery disease; LDL: low-density lipoprotein; HDL: high-density lipoprotein, GFR: glomerular filtration rate; ACEi: angiotensin converting enzyme inhibitors; ARB: angiotensin receptor blocker; PMI: periprocedural myocardial infarction; and angi angiotensin Angiotensin II receptor blockers.

**Table 2:** Comparison of the baseline clinical, demographic and laboratory data of patients with and without PMI.

	Patients Without PMI (n: 353)	Patients with PMI (n: 109)	*p*
Age (years)	61.29 ± 9.61	62.29 ± 9.51	0.341
Gender (male, %)	274 (77.6)	84 (77.1)	0.903
Body mass index (kg/m^2^)	28.47 ± 3.36	28.31 ± 3.55	0.674
Diabetes mellitus (n, %)	143 (40.5)	57 (52.3)	0.03
Hyperlipidemia (n, %)	202 (57.2)	58 (53.2)	0.460
Active smoking (n, %)	155 (43.9)	40 (36.7)	0.183
Family history of CAD (n, %)	126 (35.7)	47 (43.1)	0.161
Stroke (n, %)	11 (3.1)	2 (1.8)	0.480
ACEi or ARB use (n, %)	190 (53.8)	54 (49.5)	0.434
Beta-blockers use (n, %)	191 (54.1)	74 (67.9)	0.11
Aspirin use (n, %)	208 (58.9)	50 (45.9)	0.160
Statin use (n, %)	112 (31.7)	32 (29.4)	0.640
CCB use (n, %)	114 (32.3)	46 (42.2)	0.057
Left ventricular ejection fraction (%)	56.38 ± 4.51	55.65 ± 4.69	0.164
Hemoglobin (g/dL)	13.33 ± 1.77	12.99 ± 1.78	0.109
White blood cell count (10^9^/L)	8.93 ± 2.76	9.63 ± 3.09	0.037
Platelet count (10^9^/L)	244.5 (194.5–292)	238.5 (195–273.5)	0.612
Baseline GFR (mL/min/1.73 m^2^)	91.98 ± 21.94	82.36 ± 20.13	0.001
Baseline creatinine (mg/dL)	0.88 (0.73–1.00)	0.91 (0.79–1.01)	0.017
Total cholesterol (mg/dL)	174.85 ± 45.97	202.6 ± 72.44	0.008
LDL cholesterol (mg/dL)	107 (82–134)	99.75 (81–130)	0.769
HDL cholesterol (mg/dL)	38 (33–44)	34.5 (31–46)	0.464
Triglyceride (mg/dL)	155.5 (112–242)	167 (135–287)	0.133
Glucose (mg/dL)	146.16 ± 73.39	166.71 ± 80.79	0.044
Hba1c (%)	6.93 ± 2.12	8.93 ± 2.46	0.003
C-reactive protein (mg/L)	4.89 (2.12–11.92)	4.95 (3.02–10.85)	0.694
Three-vessel disease (n, %)	31 (8.8)	28 (25.7)	<0.001
Complex PCI (n, %)	23 (7.8)	38 (40.4)	<0.001
Proximal lesion	157 (54.5)	69 (73.4)	0.001
SYNTAX score	9 (6–12)	22.25 (14.5–28)	<0.001
Pressure (atm)	17.92 ± 1.41	18.14 ± 1.19	0.467
Number of stents	1.27 ± 0.5	1.72 ± 0.74	<0.001
Stent diameter (mm)	3.01 ± 0.38	3.13 ± 0.43	0.009
Stent length (mm)	24.93 ± 9.22	29.88 ± 10.2	<0.001
Non-Dipping pattern (n, %)	164 (46.5)	79 (72.5)	<0.001
Dipping pattern (n, %)	189 (53.5)	30 (27.5)	<0.001

PMI: periprocedural myocardial infarction; CAD: coronary artery disease; LDL: low-density lipoprotein; HDL: high-density lipoprotein, GFR: glomerular filtration rate; ACEi: angiotensin converting enzyme inhibitors; ARB: angiotensin receptor blocker; CCB: calcium channel blockers; and angi angiotensin Angiotensin II receptor blockers.

**Table 3:** Multivariate logistic regression analysis to determine the independent predictors of PMI.

Variable	*p*	Odds Ratio	95% CI	
			Lower	Upper
Non-dipping pattern	0.002	25.986	3.157	213.923
Stent length	0.016	1.148	1.025	1.285
Diabetes mellitus	0.044	6.493	1.055	40
White blood cell	0.305	1.062	0.893	1.436
Proximal lesion	0.07	5.848	0.864	40
Stent diameter	0.185	3.237	0.569	18.407
Number of stents	0.016	5.744	1.377	23.957
Aspirin	0.13	0.298	0.062	1.430
Total cholesterol	0.063	1.015	0.999	1.030
Complex PCI	0.029	9.615	1.267	71.428
Creatinine	0.198	4.518	0.455	44.829

PMI: periprocedural myocardial infarction; PCI: Percutaneous coronary intervention.

## Data Availability

The data can be obtained from the corresponding author upon request.
